# Mirrored Prominent Deck B Phenomenon: Frequent Small Losses Override Infrequent Large Gains in the Inverted Iowa Gambling Task

**DOI:** 10.1371/journal.pone.0047202

**Published:** 2012-10-16

**Authors:** Ching-Hung Lin, Tzu-Jiun Song, Yu-Kai Lin, Yao-Chu Chiu

**Affiliations:** 1 Department of Psychology, Soochow University, Taipei, Taiwan; 2 Laboratory of Integrated Brain Research, Department of Medical Research & Education, Taipei Veterans General Hospital, Taipei, Taiwan; 3 Biomedical Electronics Translational Research Center, National Chiao Tung University, Hsinchu, Taiwan; 4 Biomedical Engineering Research and Development Center, China Medical University Hospital, Taichung, Taiwan; National Research & Technology Council, Argentina

## Abstract

Since Bechara *et al.* pioneered its development, the Iowa Gambling Task (IGT) has been widely applied to elucidate decision behavior and medial prefrontal function. Although most decision makers can hunch the final benefits of IGT, ventromedial prefrontal lesions generate a myopic choice pattern. Additionally, the Iowa group developed a revised IGT (inverted IGT, *iIGT*) to confirm the IGT validity. Each *iIGT* trial was generated from the trial of IGT by multiplying by a “−” to create an inverted monetary value. Thus, bad decks A and B in the IGT become good decks iA and iB in the *iIGT*; additionally, good decks C and D in the IGT become bad decks iC and iD in the *iIGT*. Furthermore, IGT possessed mostly the gain trials, and *iIGT* possessed mainly the loss trials. Therefore, IGT is a frequent-gain–based task, and *iIGT* is a frequent-loss–based task. However, a growing number of IGT-related studies have identified confounding factors in IGT (i.e., gain-loss frequency), which are demonstrated by the prominent deck B phenomenon (PDB phenomenon). Nevertheless, the mirrored PDB phenomenon and guiding power of gain-loss frequency in *iIGT* have seldom been reexamined. This experimental finding supports the prediction based on gain-loss frequency. This study identifies the mirrored PDB phenomenon. Frequent small losses override occasional large gains in deck iB of the *iIGT*. Learning curve analysis generally supports the phenomenon based on gain-loss frequency rather than final outcome. In terms of *iIGT* and *simple versions of iIGT*, results of this study demonstrate that high-frequency loss, rather than a satisfactory final outcome, dominates the preference of normal decision makers under uncertainty. Furthermore, normal subjects prefer “no immediate punishment” rather than “final reward” under uncertainty.

## Background

Bechara *et al.* [1] designed the Iowa Gambling Task (IGT) in 1994 to simulate real-life decisions and verify decision patterns between subjects with ventromedial prefrontal (VMPFC) lesions (who suffered social function deficits) and normal controls. Serial findings of the Iowa group indicated that normal decision makers can support the Somatic Marker Hypothesis (SMH) proposed by Damasio *et al*. [2], [3]. The SMH postulates that subjects with VMPFC lesions are myopic to future consequences of their performance in real-life decision making; meanwhile, normal subjects can predict the ultimate benefits using an intact somatic system [4], [5]. In the recent decade, the IGT has been an effective means of evaluating the decision function between normal decision makers and neuropsychological patients in clinical assessment [6], [7].

In IGT, decks A and B cause subjects to lose $250 over an average of ten trials, explaining why the two decks were defined as “bad decks”. Decks C and D cause subjects to gain $ 250 over an average of ten trials, explaining why the two decks were named “good decks” [1], [8]. In the original IGT, bad deck A and good deck C displayed 10 gains and 5 losses, while bad deck B and good deck D displayed 10 gains and 1 loss [1] (Table 1, first circle of 10 trials in the IGT). In sum, the original IGT was a frequent gain based game under uncertainty. Bechara *et al.* found that subjects with affective deficits preferred bad decks A and B to good decks C and D, while normal control subjects preferred good decks C and D to bad decks A and B [4], [9], [10], [11], [12], [13].

**Table 1:** The gain-loss structure in the original IGT.

IGT	A	B	C	D
1	100	100	50	50
2	100	100	50	50
3	100, **−150**	100	50, **−50**	50
4	100	100	50	50
5	100, **−300**	100	50, **−50**	50
6	100	100	50	50
7	100, **−200**	100	50, **−50**	50
8	100	100	50	50
9	100, **−250**	100, **−1250**	50, **−50**	50
10	100, **−350**	100	50, **−50**	50, **−250**
**Final Outcomes**	**−250**	**−250**	**250**	**250**
**Gain-loss Frequency**	**10 gains**	**10 gains**	**10 gains**	**10 gains**
	**5 losses**	**1 loss**	**5 losses**	**1 loss**

Additionally, Bechara *et al.* [14] devised an inverted version of IGT (*iIGT*) to validate IGT and SMH. In the *iIGT*, Bechara *et al.* reversed the sign (+/−) of each trial in the original IGT (see Table 1) such that decks A and B have positive final outcomes ($ +250), while decks C and D have negative final outcomes ($ −250) over an average of ten trials (see Table 2). That study renamed inverted deck A as deck G, deck B as deck E, deck C as deck F, and deck D as H (for easy comparison and description, deck G is referred to hereinafter as iA; deck E as iB; deck F as iC; deck H as iD) (Table 2, first circle of ten trials in the *iIGT*).

**Table 2:** The gain-loss structure in the inverted IGT.

*iIGT*	iA	iB	iC	iD
1	**−100**	**−100**	**−50**	**−50**
2	**−100**	**−100**	**−50**	**−50**
3	**−100**, 150	**−100**	**−50**, 50	**−50**
4	**−100**	**−100**	**−50**	**−50**
5	**−100**, 300	**−100**	**−50**, 50	**−50**
6	**−100**	**−100**	**−50**	**−50**
7	**−100**, 200	**−100**	**−50**, 50	**−50**
8	**−100**	**−100**	**−50**	**−50**
9	**−100**, 250	**−100**, 1250	**−50**, 50	**−50**
10	**−100**, 350	**−100**	**−50**, 50	**−50**, 250
**Final Outcomes**	**250**	**250**	**−250**	**−250**
**Gain-loss Frequency**	**5 gains**	**1 gain**	**5 gains**	**1 gain**
	**10 losses**	**10 losses**	**10 losses**	**10 losses**

In the *iIGT*, the four decks have final outcomes opposite to those in the original IGT; the gain-loss structure is inverted as well. Decks iA and iC in the *iIGT* have 5 gains and 10 losses, while decks iB and iD have 1 gain and 10 losses (Table 2). Additionally, the *iIGT* demonstrates that gain-loss frequency and monetary value between good and bad decks inverted completely from those of the original IGT. In sum, *iIGT* is a frequent loss based game under uncertainty. However, the *iIGT* can still predict the effect of final outcome [14]. Namely, normal decision makers can gradually infer the ultimate benefits and learn to select good decks iA and iB, while also avoiding the selection of bad decks iC and iD. In short, decision makers are not attracted to the frequent large gains of decks A and B in the IGT; in addition, decision makers are not put off by frequent large losses (pains or punishments) of decks iA and iB in the *iIGT*. The Iowa group suggests that long-term outcome largely determines choice behavior under uncertainty.

However, numerous IGT studies have identified a strange phenomenon recently [15], [16], [17], [18], [19], [20], [21], [22], [23], [24], [25], [26], specifically the “prominent deck B” (PDB) phenomenon [27]. This phenomenon demonstrates that normal subjects prefer bad deck B almost as much as good deck D in the original IGT. In short, the gain-loss frequency dominates decision-makers' choices in the IGT [28], [29].

By using a simplified version of the IGT (AACC and BBDD), Lin *et al.* [27] verified the PDB phenomenon, indicating that bad deck B is selected nearly as frequently as good deck D in the BBDD version. They also suggested that deck C is preferred over deck A in the AACC version. However, this preference results in the AACC version not from final outcome, yet rather from the unbalanced gain-loss frequency between decks A (5 gains, 5 losses) and C (5 gains, 5 draws) in terms of net value in each trial (Table 3). Bechara (Sevy *et al.* and Johnson *et al.*) [30], [31] recently identified the PDB phenomenon in their experimental data. In their studies [30], subjects chose bad deck B (the mean number of card selection for deck B is “31” trials; this number is observed from the table of Sevy *et al.*, 2007) over the other three decks. Deck B was selected nearly twice as frequently in this study than it was in 1994 (the mean number of card selection for deck B is approximately “17” trials; this number is estimated based on the figure of Bechara *et al.*, 1994). Wilder *et al.* (1998) [16] mentioned the dominating influence of gain-loss frequency in the IGT [15], [16], [17], [18], [19], [20], [21], [22], [23], [24], [25], [26]. In particular, frequent gains override large losses in the bad final-outcome deck B. The PDB phenomenon is consistent with findings of the literature.

**Table 3:** The net value of each trial in the original IGT.

*iIGT*	iA	iB	iC	iD
1	100	100	50	50
2	100	100	50	50
3	**−50**	100	**0**	50
4	100	100	50	50
5	**−200**	100	**0**	50
6	100	100	50	50
7	**−100**	100	**0**	50
8	100	100	50	50
9	**−150**	**−1150**	**0**	50
10	**−250**	100	**0**	**−200**
**Final Outcomes**	**−250**	**−250**	**250**	**250**
**Gain-loss Frequency**	**5 gains**	**9 gains**	**5 gains**	**9 gains**
	**5 losses**	**1 loss**	**5 draws**	**1 loss**

Traditional analysis methods largely used the two-category format (good vs. bad) to present data, explaining their insufficiency in evaluating the effect induced by the gain-loss frequency [6], [32]. Furthermore, this study surveyed nine previous studies, which utilized *iIGT* as the research instrument (Table 4) [13], [14], [31], [33], [34], [35], [36], [37], [38]. These studies presented their data using the two-category format (good (iA+iB) *vs.* bad (iC+iD)) [13], [14], [31], [33], [34], [35], [36], [37], [38], [39]. Therefore, the mean number of each deck cannot be observed directly and the evaluation of the frequency effect is inaccessible. However, there have four studies have touched the issue of frequency effect in some *iIGT* situation. Geurts *et al.* (2006), Huizenga *et al.* (2007), Fernie (2007) and Fum *et al.* (2008) studies [28], [40], [41], [42] incorporated the four-deck format to present their data [28], [40], [41], [42]. However, Geurts *et al.* (2006), Huizenga *et al.* (2007) studies utilized the modified *iIGT* (i.e. the hungry donkey version, HDV [39]). The HDV was modified by the Crone et al. (2003) [39] and mainly aimed to explore the developmental issues of decision-making. The HDV used the donkey choices to knock the (four) doors (as decks) as the main scenario and utilized the apple number to represent the monetary value in the original *iIGT*. So, the simplified HDV may not be totally comparable to the original *iIGT*. Furthermore, while using the original *iIGT* (Bechara *et al.*, 2000), the doctoral dissertation of Fernie demonstrated the mean chosen number of four decks. However, Fernie's dissertation [42] focused on other manipulations (e.g., probabilistic version) in *iIGT*. Notably, Fum et al. (2008) directly demonstrated the frequency effect override the final-outcome in the standard *iIGT*. Consequently, Geurts *et al.* (2006), Huizenga *et al.* (2007), Fernie (2007) and Fum *et al.* (2008) had revealed the partial effect of gain-loss frequency in these modified *iIGT*. However, the mirrored PDB phenomenon was not clearly defined before as well as illustrated and discussed in detail. Therefore, this work used *iIGT* and, in particular, the two simple versions of *iIGT* with extended stage to verify the observations of Fum *et al.* (2008), Fernie (2007), Geurts *et al.* (2006) and Huizenga *et al.* (2007).

**Table 4:** Summary of presentation methods from thirteen *iIGT* studies.

Research Groups	Subjects	Presentation Format
Bechara *et al.* (2000)	Controls: 20 subjects (8 males, 12 females); Age: 22∼68 (42.1±11.6) years old; Education: 14.3±1.2 years	Two-category format
Bechara *et al.* (2002)	Controls: Impaired: 9 subjects (2 males, 7 females); Age: 44.7±7.2 years old; Education: 14.7±1.7 years; Non-impaired: 22 subjects (12 males, 10 females); Age: 41.2±8.9 years old; Education: 15.3±2.3 years	Two-category format
Crone *et al.* (2003)	Three groups: (1) 27 subjects (12∼13 years old); (2) 28 subjects (15∼16 years old); (3) 35 subjects (18∼25 years old)	Two-category format
Davis *et al.* (2008)	452 subjects (285 males, 167 females); Age: 25∼50 (men 34.51±7.7, women 34.31±7.33) years old; Education: seven categories	Two-category format
Desmeules *et al.* (2008)	126 adult women; Age: 18∼75 (43.03±16.34) years old; Four groups: (1) low BIS/low BAS; (2) high BIS/low BAS; (3) low BIS/high BAS; (4) high BIS/high BAS	Two-category format
Dretsch *et al.* (2007)	35 subjects (10 males, 25 females); Age: 22.7±5.07 years old; Two groups: (1) low working memory; (2) high working memory	Two-category format
Geurts *et al.* (2006)	Controls: 22 subjects (18 males, 4 females); Age: 10.0±1.3 years old; IQ: 108.9±16.2	Four-deck format
Fum *et al.* (2008)	27 subjects (about 17 males); Age: 20∼31 (21.4±2.5) years old	Four-deck format
Huizenga *et al.* (2007)	Four groups: (1) 61 subjects (33 boys, 28 girls, 6∼9 years old); (2) 61 subjects (27 boys, 33 girls, 10∼12 years old); (3) 59 subjects (29 boys, 30 girls, 13∼15 years old); (4) 61 subjects (12 males, 49 females, 18∼25 years old) (Reanalysis of Crone and Van der Molen (2004)) [55]	Four-deck format
Johnson *et al.* (2008)	Controls: 87 subjects who were teetotalers (37 males, 50 females); Age: 16.11±0.52 years old; Four groups: (1) teetotalers; (2) occasional-drinkers; (3) consumed alcohol during the past 30 days; (4) binge-drinkers	Two-category format
Kim *et al.* (2006)	Controls: normal controls without a history of behavioral disorder 30 subjects (males); Age: 39.1±7.3 years old; Education: 14.0±1.8 years; Four groups: (1) alcohol-dependent patients with history of behavioral disorder (AD CD+); (2) alcohol-dependent patients without history of behavioral disorder (AD CD−); (3) normal controls with history of behavioral disorder (NC CD+); (4) normal controls without history of behavioral disorder (NC CD−)	Two-category format
Must *et al.* (2006)	Controls: 20 subjects (11 females, 9 males); Age: 42.5±10.7 years old; Education: 15.1±9.4 years; Two groups: (1) control; (2) depression	Two-category format
Windmann *et al.* (2006)	22 subjects (11 males, 11 females); Age: 26.3±6.66 years old	Two-category format

Based on two experiments, this study illustrates the mirrored PDB phenomenon in *iIGT*. While focusing on the four-deck format to present the data, the first experiment attempted to reproduce the original findings of the *iIGT*. Based on the experimental paradigm of Lin *et al.* (2007) [27], the second experiment enrolled two simplified versions of *iIGT* (iA-iA-iC-iC; iB-iB-iD-iD) to illustrate the frequency effect in the context of *iIGT*.

In the first experiment, if gain-loss frequency could predict decision-making behavior in the *iIGT*, subjects should avoid selecting high-frequency loss decks iB and iD as well as prefer related frequent-gain decks iA and iC (Table 2). Namely, the mirrored PDB phenomenon should be observable. Conversely, if the final outcome is dominant, subjects should prefer good final-outcome decks iA and iB to bad final-outcome decks iC and iD. Thus, the mirrored PDB phenomenon should not occur.

In particular, evaluating the two perspectives (gain-loss frequency vs. final-outcome) focuses on evaluating the mean number of good deck iB (Table 2). If the preference of most participants for good deck iB equals that for good deck iA, then the experimental outcome obtained by Bechara *et al.* [14] is replicated and final outcome applied. In contrast, if good deck iB is chosen less frequently than good deck iA or nearly as frequently as bad decks iC and iD, the gain-loss frequency is a valid means of predicting the choice pattern under uncertainty.

Based on the simplified version (iA-iA-iC-iC and iB-iB-iD-iD) of the *iIGT*, the second experiment aims to verify the findings of Lin *et al.* (2007) [27]. This experiment also further evaluates the predictive power of the final outcome and gain-loss frequency in relatively uncomplicated situations. If the final outcome is predictive, decision-makers should prefer good deck iA over bad deck iC in the iA-iA-iC-iC version of the *iIGT*. Contrastly if gain-loss frequency is predictive, decision makers should prefer good deck iA (5 gains, 10 losses) nearly equal to bad deck iC (5 gains, 10 losses) (Table 2) [43]. Nevertheless, based on the explanation of Lin *et al.* (2007) as well as the illustrated example of Chiu and Lin (2007) [43], the subjects' preference for deck iA (5 gains) over deck iC (5 draws) can also be interpreted as the imbalanced gain frequency in terms of net value calculation (Table 5).

**Table 5:** The net value of each trial in the inverted IGT.

*iIGT*	iA	iB	iC	iD
1	**−100**	**−100**	**−50**	**−50**
2	**−100**	**−100**	**−50**	**−50**
3	50	**−100**	0	**−50**
4	**−100**	**−100**	**−50**	**−50**
5	200	**−100**	0	**−50**
6	**−100**	**−100**	**−50**	**−50**
7	100	**−100**	0	**−50**
8	**−100**	**−100**	**−50**	**−50**
9	150	1150	0	**−50**
10	250	**−100**	0	200
**Final Outcomes**	**250**	**250**	**−250**	**−250**
**Gain-loss Frequency**	**5 gains**	**1 gain**	**5 draws**	**1 gain**
	**5 losses**	**9 losses**	**5 losses**	**9 losses**

Furthermore, if final-outcome has a predictive value, decision makers should significantly prefer good deck iB ($ +250) over bad deck iD ($ −250) in the iB-iB-iD-iD version of *iIGT*. Conversely, if gain-loss frequency has a predictive value, decision-makers should have no strong preference for high-frequency loss decks iB (1 gain, 10 losses) versus iD (1 gain, 10 losses) (Table 2). Therefore, each simplified version of *iIGT* is an index of verifying the guiding power of final-outcome and gain-loss frequency in a relatively simple context. While this study evaluates the guiding power of two factors (gain-loss frequency vs. final-outcome) under the frequent loss based circumstances (*iIGT* and simple version *iIGT*), thoroughly elucidating decision behavior under uncertainty should be of priority concern.

## Materials and Methods

### Participants

#### Experiment 1: *iIGT*

The first experiment enrolled 48 volunteers who completed the computer version of the *iIGT*. Age effect and gender difference were controlled (first experiment (*iIGT*): 24 females, mean age = 19.92 years, SD = 1.21; 24 males, mean age = 20.42 years, SD = 1.38). In the present study, the undergraduates were free to participate in any psychological experiment in the university, including the computer game and who participated in return for course credit [23], [44], [45], [46]. This study procedure was consistent with publicly available literature. After completing the whole game, the authors provided a two hours verbal statement in regard to the testing purpose and psychological mechanism of the computer game for all subjects. This data was collected about the 2007, the Institutional Review Board approval and written informed consent form in Soochow University were not available during that time (the IRB for human study was constructing and written informed consent form was available after 2009 in Soochow University). This study was conducted in accordance with the unwritten rule of the Taiwan Psychological Society. Accordingly, the data were analyzed in group level and reported anonymously. Further, the IGT was designed to simulate the real-life decision; so it looked like a general computerized card-game on the internet. Many research websites provided some internet versions to recruit the online subjects (such as: http://www.millisecond.com/download/samples/v3/IowaGamblingTask/IowaGamblingTask.web; http://pebl.sourceforge.net/battery.html), who can take part in the internet version of IGT totally free.

#### Experiment 2: simple version of the *iIGT*

The second experiment recruited another 48 volunteers who completed the simple version of the *iIGT*. The age and gender effects were balanced by using 24 females (mean age = 20.29 years, SD = 1.20) and 24 males (mean age = 20.58 years old, SD = 1.21). Each simple version of *iIGT* was performed by 12 female and 12 male participants. The gender difference and subject age were generally controlled. The procedure of subject recruitment is equivalent to the **Experiment 1**.

### Task

#### Experiment 1: *iIGT*

The gain-loss structure of the *iIGT* used in this study was that with inverted signs (+/−) developed by Bechara *et al.* (1994) original IGT table (see Table 1). The card position was balanced using the Latin-square design (24 card arrangements: iA-iB-iC-iD, iA-iC-iD-iB, iA-iD-iB-iC………, iD-iC-iB-iA) to prevent the visual field and card position confounding the card selection. Each card arrangement was applied to two participants (one female and one male) to ensure gender balance. Additionally, the computer versions of *iIGT* and data-analysis programs were programmed with Matlab 7.0. for data recollection and analysis. The original instructions for the IGT [14] were administered to each participant in the *iIGT* and simplified *iIGT*. Each subject played the two-stage *iIGT* (100 trials, 100 trials) to clarify their extended preference for these decks.

#### Experiment 2: simple version of the *iIGT*

The gain-loss structures of two simple versions (iA-iA-iC-iC and iB-iB-iD-iD) of the *iIGT* were generated from that of *iIGT* (Table 2). Also, Matlab 7.0 was utilized here to computerize simple version of *iIGT* and analysis-programs for recording the selection behavior and post game analysis. In each simple version of *iIGT*, the card positions were counterbalanced (e.g. iA-iA-iC-iC, iA-iC-iA-iC…) and experimental procedures for the original IGT were mostly followed [14].

### Procedure

Each participant was free to register a time slot at the laboratory for a behavioral experiment and subjects were asked to play a four-card computer game twice. When each participant came to the laboratory, the experimenter showed them the original introduction of IGT [14] for reading. Furthermore, the experimenter confirmed that each participant understood the rules of the game. After completing the first stage, participants were instructed to play the game once more, and the experimenter highlighted that the internal rules of the second stage were the same as those of the first stage. The mean number and probability maps of card selection, learning curve of each deck and linear regression of probability and monetary value were implemented for description and inference statistics. The general linear model: repeated measurement was taken to test the influence of two factors (final-outcome [(iC+iD) vs. (iA+iB)] *vs.* gain-loss frequency [(iA+iC) vs. (iB+iD)]). Also, the one way ANOVA was conducted to detail the differences of decks (iA-iB-iC-iD) and evaluate the learning effect, i.e. testing the difference between each of the two blocks (block: each 20 trials) in each deck. Furthermore, exactly how the two stages of each deck differ was evaluated using a paired sample T-test.

## Results

### Experiment 1: *iIGT*

**Figure 1:**
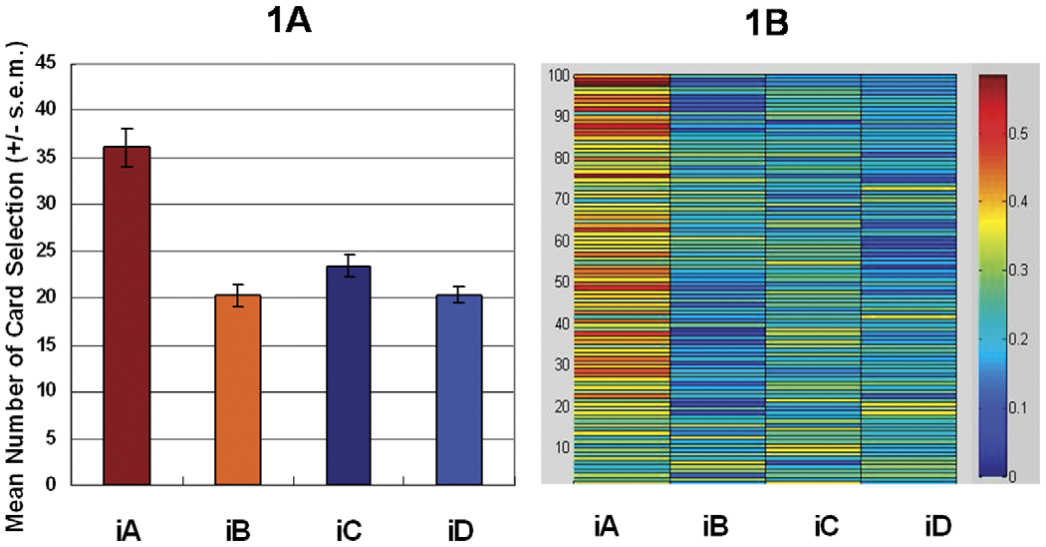
Mean deck preference and selection probability during the first stage of the *iIGT*. (1a) The cumulative number of each deck in the first stage of the *iIGT* demonstrates that subjects strongly preferred good deck *iA* to the other three decks. However, normal decision-makers should not have avoided good deck iB; this experimental result is inconsistent with the basic assumption in the IGT. Notwithstanding, if the net score of good decks (iA+iB) is used to substrate that of bad (iC+iD) decks, the basic assumption in the IGT still holds. (1b) Warm colors represent high selection probability and cold colors represent low selection probability. Probability analysis across 100 trials indicated that most subjects preferred to choose the good deck iA and avoided choosing good deck iB.

**Figure 2:**
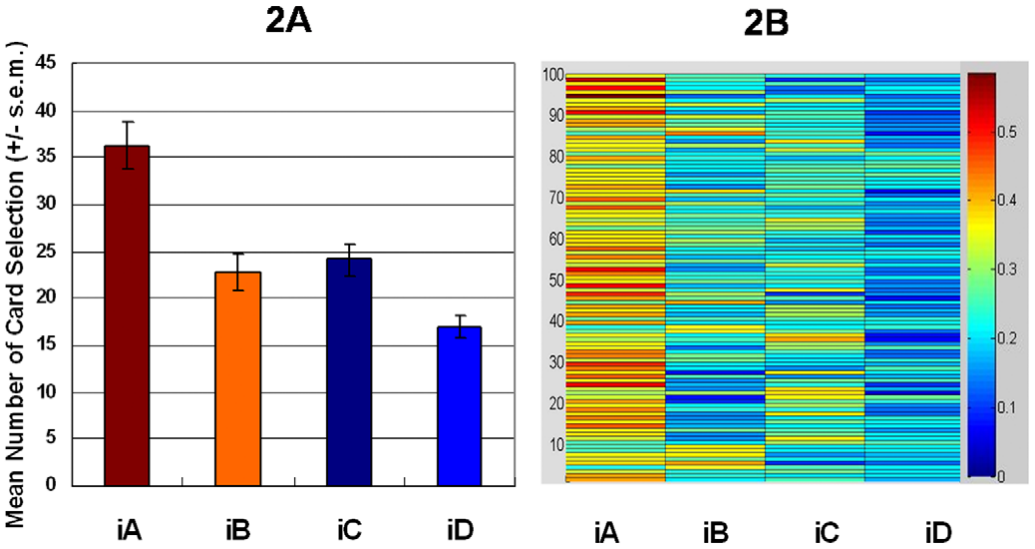
Mean deck preference and selection probability during the second stage of the *iIGT*. (2a) The second stage of the *iIGT* generally replicated the findings of the first stage. The mean number of selecting deck *iA* significantly exceeds that for the other three decks in most cases (Table 6). The experimental results obtained for “sunken deck iB” clearly support the PDB phenomenon in some IGT studies. (2b) The warm color indicates a high likelihood of selection while the cold color indicates a low likelihood. Even in the second stage of *iIGT*, most subjects still preferred good deck iA and avoided good deck iB.

The experimental results demonstrated that subjects favored deck iA over the other three decks in the first and second stages (Figures 1 and 2) [27], [29], [47]. The mirrored PDB phenomenon was confirmed in the present study. The general linear model: repeated measurement (two-factors: final-outcome [(iC+iD) vs. (iA+iB)] *vs.* gain-loss frequency [(iA+iC) vs. (iB+iD)]) was applied to test the *iIGT*. The primary effects of final outcome (first stage, *F* (1, 47) = 17.91, *p*<.01; second stage, *F* (1, 47) = 19.06, *p*<.01) and gain-loss frequency (first stage, *F* (1, 47) = 29.47, *p*<.01; second stage, *F* (1, 47) = 18.91, *p*<.01) were all significant (Figures 1 and 2). The interaction between the two factors was significant only in the first stage (first stage, *F* (1, 47) = 13.31, *p*<.01; second stage, *F* (1, 47) = 2.31, *p* = .14).

Additionally, the one-way ANOVA (iA-iB-iC-iD) and *post hoc* analysis was performed to double check and evaluate the difference between each of the two decks.

However, detailed analysis reveals the most significant effect as the difference between deck iA and the other three decks (iB, iC, iD). Table 6 lists the experimental result of a post hoc analysis (corrected with Tukey HSD) for each pair of decks in two stages.

**Table 6:** Post hoc analysis (Tukey HSD) for each two-deck in the *iIGT.*

Stage 1	Mean Difference	P	Stage 2	Mean Difference	P
A–B	15.85	0.00 **	A–B	13.42	0.00 **
A–C	12.67	0.00 **	A–C	12.13	0.00 **
A–D	15.81	0.00 **	A–D	19.29	0.00 **
B–C	−3.19	0.40	B–C	−1.29	0.96
B–D	−0.04	1.00	B–D	5.88	0.12
C–D	3.15	0.41	C–D	7.17	0.04 *

*Note:*

** *p<.01,*

* *p<.05.*

**Figure 3:**
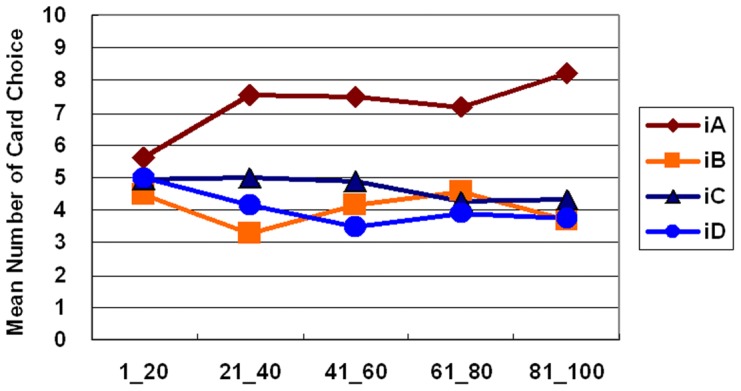
Mean number of cards selected in blocks during the first stage in the *iIGT*. The learning curve for the first stage of the *iIGT* clearly shows the differences between the four decks. Subjects gradually increased their preference for deck *iA* over the other three decks. However, final outcome ($ 250) or gain-loss frequency (5 gains, 5 losses) explained participant preference for deck *iA*. Conversely, avoidance of good deck *iB* ($ 250) can only be explained by gain-loss frequency (1 gain, 9 losses).

**Figure 4:**
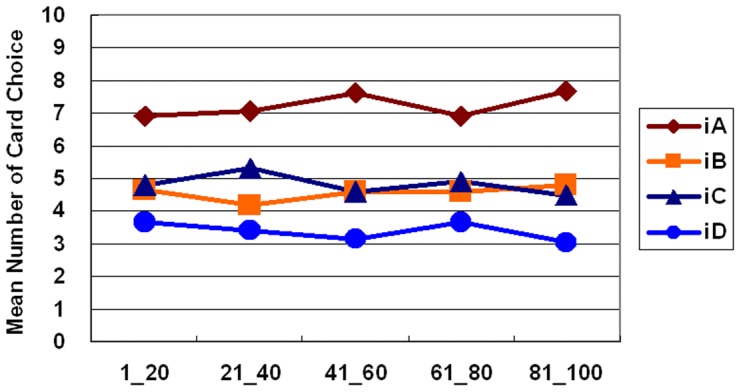
Mean number of cards selected in blocks during the second stage of the *iIGT*. According to the learning curve for the second stage, subject preference for these four decks remained steady. The main effect of blocks was generally insignificant. Even in the second stage, subjects did not fully infer the final benefit of deck iB.

Furthermore, the learning curve for the both stage shows that subjects clearly preferred the good deck iA, but avoided choosing the other three decks throughout the game (Figures 3 and 4). One-way ANOVA was initiated to test the learning effect with each 20 trials of block in each deck. Block significantly affected decks iA and iD in the first stage (deck iA: *F* (4, 235) = *2.71*, *p*<.05; deck iD: *F* (4, 235) = *2.93*, *p*<.05), but not the second stage. Post hoc analysis revealed a significant difference only between blocks one and five in deck iA (mean differences = −2.63, *p*<.05) and between blocks one and three in deck iD (mean differences = 1.50, *p*<.05). Nevertheless, no significant learning effect existed in decks iB and iC in either stage. On the other hand, the block-by-block comparison between the good and bad decks (iA vs. iC; iB vs. iD) indicated that good deck iA was significantly preferred over bad deck iC in most blocks of the game; however, good deck iB was not significantly preferred over bad deck iD in most blocks of the first and second stages (Tables 7 and 8).

**Table 7:** Paired-t test for the comparable decks in five blocks of first-stage *iIGT*.

Block	t	df	P	Block	t	df	P
A1–C1	1.20	47	0.24	B1–D1	−1.38	47	0.17
A2–C2	2.72	47	0.01 *	B2–D2	−1.79	47	0.08
A3–C3	2.52	47	0.02 *	B3–D3	1.24	47	0.22
A4–C4	4.21	47	0.00 **	B4–D4	1.07	47	0.29
A5–C5	4.06	47	0.00 **	B5–D5	−0.08	47	0.94

*Note:*

** *p<.01,*

* *p<.05.*

**Table 8:** Paired-t test for the comparable decks in five blocks of second-stage *iIGT.*

Block	t	df	P	Block	t	df	P
A1–C1	2.32	47	0.03 *	B1–D1	1.66	47	0.10
A2–C2	1.80	47	0.08	B2–D2	1.06	47	0.30
A3–C3	3.44	47	0.00 **	B3–D3	2.19	47	0.03 *
A4–C4	2.27	47	0.03 *	B4–D4	1.32	47	0.20
A5–C5	3.93	47	0.00 **	B5–D5	2.63	47	0.01 *

*Note:*

** *p<.01,*

* *p<.05.*

To monitor the learning effect between the two stages in each deck, a paired sample T- test was performed to explore the stage difference. The analysis indicated that only decks iB and iD differed significantly between the two stages (deck iA: *t*(47) = −0.1, *p* = .92; deck iB: *t*(47) = −2.02, *p*<.05; deck iC: *t*(47) = −.58, *p* = .56; deck iD: *t*(47) = 3.21, *p*<.01). This finding revealed that the stage learning effect was restricted to high-frequency loss decks. A new launched analysis to disclosure the correlation between monetary value and consecutive choice for each deck was depicted in supplementary material (Figure S1).

### Experiment 2: Simple version of *iIGT* (iA-iA-iC-iC)

**Figure 5:**
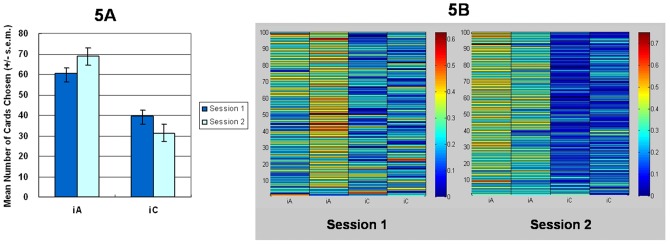
Mean deck preference and choice probability maps during the two stages in the simple (*iA*-*iA*-*iC*-*iC*) version of the *iIGT*. (5a) In the simplified *iIGT*, decision-makers preferred deck *iA* over deck *iC*. However, good deck *iA* ($ 250) contains good final outcome and a better high-frequency gain (5 gains, 5 losses) than bad deck *iC* ($ −250; 5 draws, 5 losses). Therefore, the gain-loss frequency and final outcome can be used to interpret the experimental result for the *iA*-*iA*-*iC*-*iC* version. Distinguishing the guiding principle for choice is difficult in this version. (5b) The warm color represents high selection probability and the cold color represents low selection probability. The selection probability maps demonstrated that most subjects preferred to choose good deck iA and avoided bad deck iC across both stages.

The empirical result for the simple version (iA-iA-iC-iC) of the *iIGT* identified predicted final outcome, indicating that most subjects preferred deck iA to iC in both stages (Figure 5A). The probability maps of each trial revealed that across the two sessions, subjects favored deck iA (Figure 5B). Good deck iA was selected more frequently than bad deck iC (first stage, *t* (23) = 3.71, *p*<.01; second stage, *t* (23) = 4.55, *p*<.01). However, the present finding can also be interpreted as the effect of gain-loss frequency. This is owing to that the gain-frequency of iA exceeds that of iC (see Table 5).

**Figure 6:**
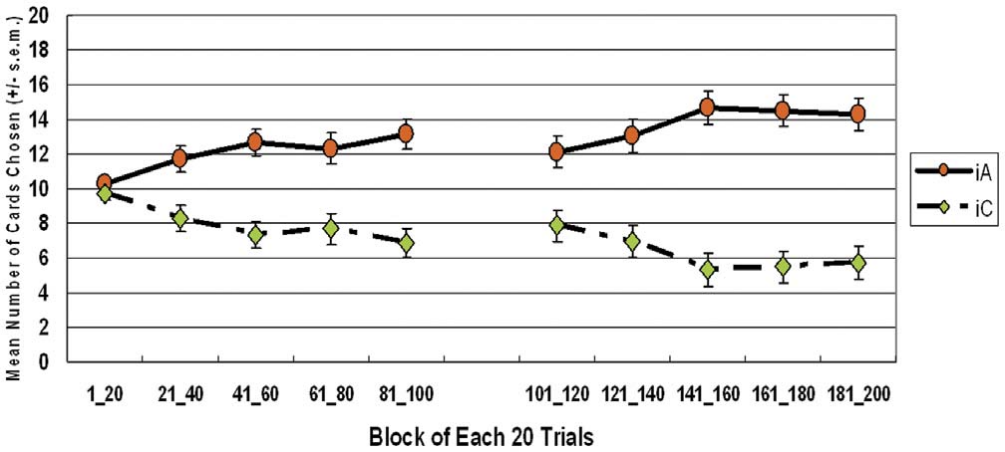
Mean number of cards selected in blocks in the simple (*iA*-*iA*-*iC*-*iC*) version of the *iIGT* during the two stages. The *iA*-*iA*-*iC*-*iC* version clearly indicates that subjects increasingly chose deck *iA* and avoided deck *iC* in both stages of the game. This experimental result coincides with the prediction of final outcome, because deck *iA* has a positive final outcome ($ 250) and deck *iC* has a negative final outcome ($ −250). However, gain-loss frequency can also explain this experimental result. According to Chiu and Lin (2007) [43], deck *iC* actually had **5 draws** and 5 losses, and was not balanced with deck *iA* (**5 gains**, 5 losses) with the calculation within each trial. Thus, subject learning tendency between stages in the *iA*-*iA*-*iC*-*iC* version can be forecast using the gain-loss frequency and final-outcome.

One-way (blocks (1–5)) ANOVA was applied to test the learning effect between two decks (iA–iC subtraction) (Figure 6). The main block effect was non-significant in each stage (first stage, *F* (4, 115) = 2.12, *p* = .08; second stage, *F* (4, 115) = 1.31, *p* = .27), although the ascending curve of deck iA and the descending curve of deck iC seemed to indicate that participants progressively identified their preference for good deck iA in both stages.

Additionally, the general linear model: repeated measurement (stage 1 (iA–iC) vs. stage 2 (iA–iC)) was enrolled to further test the learning effect on stage level, and indicated a significant difference between the two stages (*F* (1, 23) = 5.23, *p*<.05). This observation differed from the results of the first experiment of *iIGT*.

### Experiment 2: Simple version of *iIGT* (iB-iB-iD-iD)

**Figure 7:**
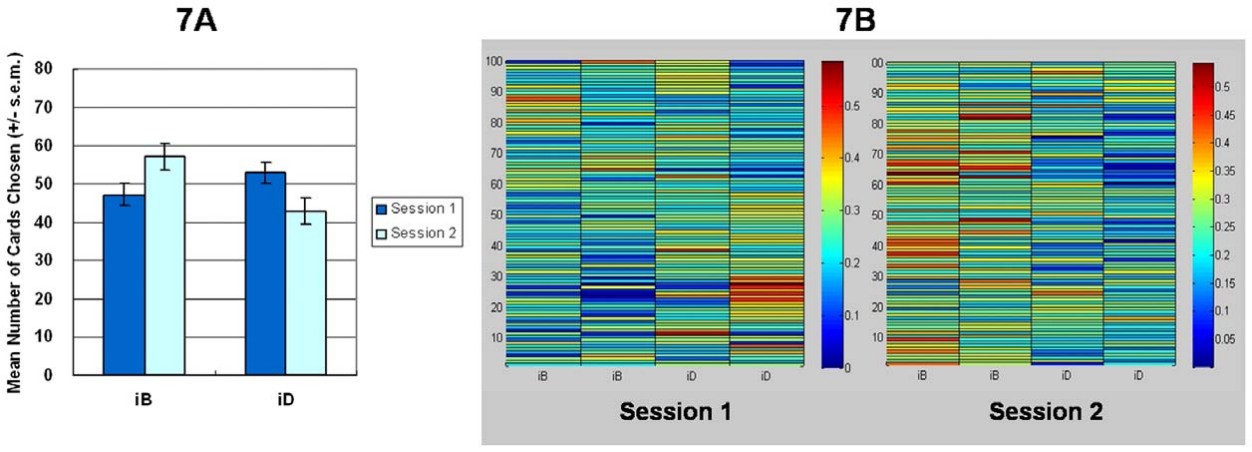
Mean deck preference during both stages in the simple (*iB*-*iB*-*iD*-*iD*) version of the *iIGT*. (7a) Empirical data indicates that good deck *iB* ($ 250) and bad deck *i*D ($ −250) were chosen a roughly equal number of times. The experimental result for the *iB*-*iB*-*iD*-*iD* version replicated the findings for the *iIGT*; that is, subjects had difficulty inferring the final-outcome during the two stages. Based on the primary assumption of IGT, the simple version of *iIGT* should enable subjects to easily register somatic markers for a specific target (deck). Conversely, even during the second stage, subjects were most influenced by high-frequency losses and less by final outcome of decks *iB* and *iD*. (7b) Warm color represents high probability of selection and cold color represents low probability of selection. In both stages, subjects chose bad decks iD nearly as often as good decks iB. Notably, the selection probability maps demonstrated that the red and yellow color lines (trials) were mostly located on deck iD in the first stage and shifted to deck iB during the second stage.

The observed result of the iB-iB-iD-iD version of *iIGT* verified that subjects' choice was mostly based on gain-loss frequency; contradicting the basic assumption that final outcome motivated subjects. Most subjects chose good deck iB almost as frequently as bad deck iD during the first stage (*t* (23) = −1.01, *p* = .33) and second stage (*t* (23) = 2.03, *p* = .054) (Figure 7A). The selection probability of each trial indicated that subjects displayed nearly equal preference for good deck iB and bad deck iD, nevertheless, selection patterns differed between the two stages (Figure 7B).

**Figure 8:**
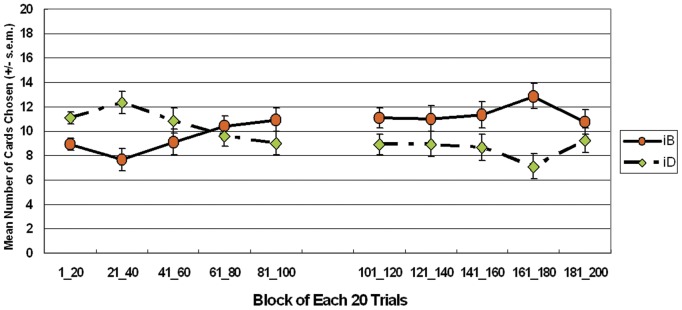
Mean number of cards selected in blocks in the simple version (*iB*-*iB*-*iD*-*iD*) of the *iIGT* during the two stages. According to the learning curve for the *iB*-*iB*-*iD*-*iD* version, subjects should generally prefer good deck *iB*; however in this simplified version of *iIGT*, the learning effect of blocks in each deck was generally non-significant. Furthermore, crossover occurred during the latter phase of the first stage. Even the two learning lines of decks *iB* and *iD* did not significantly bifurcate during the second stage. Notably, stages 1 and 2 differed significantly.

The learning curves of decks iB and iD in both stages were typically twisted together (Figure 8). One-way (blocks (1–5)) ANOVA testing was used to clarify the learning effect between two decks (iB–iD subtraction). The main effect of block cannot be observed in the iB-iB-iD-iD version in each stage (first stage, *F* (4, 115) = 2.19, *p* = .07; second stage, *F* (4, 115) = .71, *p* = .59).

However, this study performed a general linear model: repeated measurement (stage 1 (iB–iD) vs. stage 2 (iB–iD)) in the iB-iB-iD-iD version to test the effect of learning on stage level, and demonstrated a significant difference between the two stages (*F* (1, 23) = 13.64, *p*<.01). This finding is consistent with the result of the first experiment of *iIGT*. The additional investigation of testing the relationship between monetary value and consecutive choice was stated in supplementary material (Supplementary Figure 2).

## Discussion

In both experiments, the gain-loss frequency is the main predictive factor to explain the choice pattern of decision makers under uncertainty. The PDB and the mirrored PDB phenomena demonstrate that the gain-loss frequency overrides the final outcome separately in frequent-gain (IGT) and frequent-loss (*iIGT*) based circumstances. Moreover, the fact that the decision makers preferred good deck C in the IGT and avoided bad deck iC in the *iIGT* could also be interpreted as the influences of gain-loss frequency. Restated, the imbalanced gain-loss frequency (with net value calculation between bad deck A and good deck C in the IGT (Table 3) as well as between good iA and bad deck iC (Table 5)) is a serious confounding to make the two factors (gain-loss frequency vs. final outcome) interpret the results congruently. However, the PDB phenomenon in IGT and mirrored PDB phenomenon in *iIGT* can only be explained with the gain-loss frequency. Furthermore, few studies have demonstrated that the frequency effect overrides the final outcome in the simple version of *iIGT*. The second experiment confirmed previous findings based on gain-loss frequency that decision makers have difficulty in estimating the final outcome under uncertainty.

### Gain-loss frequency vs. Final-outcome

Statistical results in the first experiment indicate that two factors (final-outcome vs. gain-loss frequency) similarly influence choice behavior. However, detailed statistical analysis of behavioral data generally supports the perspective of gain-loss frequency. Specifically, the first experiment which adopted the analysis method of four-deck format did not fully replicate the original findings of Bechara *et al.* (2000) [14]. Figures 1 and 2 indicate that most subjects favor good deck iA not only over bad decks iC and iD, but also over good deck iB. Additionally, good deck iB is insignificantly preferred over bad decks iC and iD in the first experiment. The mirrored PDB phenomenon is clearly observed here (Figures 1 and 2). However, the decision makers highly preferred deck iA in *iIGT*, possibly due to the compound effect of gain-loss frequency and final outcome.

If the final-outcome is a predictive factor, as suggested by the Iowa group, good decks iA and iB should exert an equal amount of influence on choice behavior under uncertainty [1]. We posit that decision makers avoid selecting good deck iB, owing to its high-frequency loss in the *iIGT* (Table 2). Frequent losses ($ −100) of deck iB kept subjects away from this deck on a trial and error basis. Therefore, most subjects had difficulty hunching the good final-outcome of deck iB. This postulation confirms that of previous investigations, which suggest the PDB phenomenon in the original IGT [27], [48], [43].

Additionally, in the iB-iB-iD-iD version of the *iIGT*, normal decision makers chose high-frequency-loss decks iB (advantageous final-outcome) and iD (disadvantageous final-outcome) with an almost identical frequency (Figures 7 and 8). This study on the sensitivity of frequent loss is not unique. In the iA-iA-iC-iC version of the *iIGT*, subjects preferred the good deck iA rather than bad deck iC (Figures 5 and 6). Although this observation can be interpreted with a final outcome reasonably, gain-loss frequency is not predictive.

Notably, if the gain-loss frequency is a dominant factor in the original IGT, as suggested in other studies [15], [16], [23], [27], [28], [42], [43], [47], [49], [50], [51]. In the first experiment, the low-frequency loss decks iA and iC should be chosen more frequently than high-frequency loss decks iB and iD in *iIGT*. In the iA-iA-iC-iC version of the second experiment, deck iA should be chosen nearly equal to deck iC due to the same gain-loss frequency.

However, the result is also not entirely reproduced in terms of gain-loss frequency (i.e. the low-frequency loss deck iC is not chosen significantly nearly equal to deck iA) (Figures 1 and 2). Restated, the low-frequency loss decks iA and iC should be chosen to be nearly equal in the simple version of *iIGT* (iA-iA-iC-iC) (Figure 7). Nevertheless, deck iC chosen less than deck iA may be owing to the unbalanced gain-loss frequency (see Table **5**, net value of each trial: deck iA (**5 gains**, 5 losses) vs. deck iC (**5 draws**, 5 losses)). This phenomenon is also found in the original IGT [27], [43].

Chiu & Lin [27], [43] demonstrated that the gain-loss frequencies of good deck C (net value in each trial, 5 gains and **5 draws**) and bad deck A (net value in each trial, 5 gain and **5 losses**) are unbalanced in the original IGT (Table 3). That study also provided a modified IGT to balance the gain-loss frequency between decks A and C. Notably, the subjects were no longer preferred over the good deck C [27], [43].

Therefore, the preference of decision makers for good deck C over bad deck A cannot simply be attributed to guidance related to not only the final outcome, but also to gain-loss frequency. Similarly, the preference of subjects for good deck iA (net value in each trial, **5 gains** and 5 losses) over bad deck iC (net value in each trial, **5 draws** and 5 losses) in this study demonstrates the effect of both the final outcome and gain-loss frequency (Table 5). We recommend that a future study develop a modified *iIGT* with a controlled deck iC (5 gains and 5 losses, real comparable with deck iA) to illustrate the deck iC phenomenon in the *iIGT*.

Crone *et al.* [40], [41], [51] observed a similar phenomenon in their serial studies with their modified *iIGT* (i.e. hungry donkey version). Additionally, by using two-stage *iIGT* based on the gain-loss structure of Bechara *et al.* (2000) [14], Fernie (2007) and Fum et al. (2008) [28], [42] demonstrated that most subjects avoid choosing the good deck iB (deck E). Our observations for *iIGT* and the simple versions of *iIGT* (i.e. the “mirrored PDB phenomenon”) correspond to the findings of Fernie (2007) and Fum et al. (2008) [28], [42].

### Learning effect with block difference

Our results are mostly inconsistent with the suggestion of the Iowa group that decision makers can approach good final-outcome decks iA and iB during the first stage of *iIGT* [14]. In the present *iIGT* and simple version *iIGT* experiments, the block learning curve reveals few significant differences between each of the two blocks. Namely, the preference for certain decks is determined in the early blocks in the high-frequency loss of the *iIGT*. Specifically, the learning effects (approach vs. avoidance) are difficult to observe globally in both stages of both experiments (Figures 3, 4, 6, and 8). Some studies have suggested that the decision learning model under uncertainty is largely based on the gain-loss frequency rather than the expected value [6], [29], [51].

Alternatively, some research groups directly manipulated the value of final-outcome in the IGT, illustrating that subjects' choice patterns are less influenced by the change in final outcome [28], [53]. Above observations also suggested that even normal subjects are significantly influenced by frequent gain-loss and magnitude under uncertainty [28], [42], [49], [50], [54]. Also, Crone *et al.* (2004) and Stocco *et al.* (2009) [24], [50] emphasized that frequent loss is the key component guiding the choice behavior, although they used the modified IGTs. In sum, under high-frequency gain or loss situations, subject learning regarding final outcome is difficult to identify [47]. Therefore, the unstable learning effect of the *iIGT* and non-significant learning effect of the simple *iIGT*, particularly the iB-iB-iD-iD version, imply that the affective system is largely directed by the immediate perspective rather than long-term reasoning under uncertainty [29], [52].

### Learning effect with stage difference

According to the above paragraph on the learning effect on blocks, the learning effect in decks iB and iD can also be observed between stages (Figures 3 and 4). Therefore, inconsistencies arise between this study and the *iIGT* study of Bechara *et al.* (2000) [14] (learning effect was observed in first stage). Notably, if this study is illustrative of gain-loss frequency, interpreting the original findings of the *iIGT* is complex [14]. By using a series of over 100 trials, an increasing number of studies [23], [27], [29], [39], [51], [52], [55], [56] have monitored the learning effect of the IGT. Remarkably, a growing number of studies have difficulty in observing learning tendencies in standard administration (100 trials).

Alternatively, both the simple versions of the *iIGT* reveal stage differences. The subjects appear to gradually approach good final-outcome decks, especially during the second stage (Figures 6 and 8). The extended trial number and relatively simple context may be useful for observing the learning effect in terms of final outcome [57], [58]. Namely, the decreased uncertainty should make it easier for normal subjects to hunch the final outcome when using the simple version of the *iIGT*. However, the present simple versions did not display this consequence in the first stage. However, the two-stage administration (100 trials, 100 trials) provides subjects with a double opportunity to gain further insight into the final outcome. Unfortunately, according to our results, the learning effect is less robust than that suggested by the Iowa group [1], [4], [14].

This study raises two issues related to IGT research [4]. The degree of uncertainty may be an important factor that is negatively correlated with hunching state accessibility in these uncertain gambles. Some studies have discussed the effect of modulating the degree of uncertainty on choice behavior in dynamic gambling [51], [57], [58], [59], [60], [61]. This degree of uncertainty may be a significant variable in differentiating between decision systems driven by emotion and those driven by logic [62]. The trial number may be another critical aspect when assessing subject performance. Additionally, extended trial number is important in illustrating the subject performance of the *iIGT*. However, the clinical version of the IGT has been used as a clinical assessment method [63], and still possesses the standard trial number of only 100 trials. The number of trials, 100, was roughly defined at the beginning of the IGT study [1], [4]. Nevertheless, according to an increasing number of IGT related studies, 100 trials is insufficient to replicate the original findings of the Iowa group [23], [27], [29], [39], [51], [52], [55], [56].

### Correlation between monetary value and choice probability

By using correlation analysis, this study examined how monetary value and the probability of consecutive choice are related. Results of our two experiments indicated that subjects largely disregarded monetary value when facing uncertainty (Figures S1 and S2). The coefficient of determination (R-square) in each deck confirmed that the monetary value was unnoticed or quickly forgotten by subjects following the next trial. This phenomenon implies that when subjects were situated in uncertain circumstances, their choice behavior was dominated by the gain-loss sign (+/−); in addition, they disregarded the value variation in each deck. Therefore, the large gains in certain decks did not cause subjects to increase their preferences for those decks in the following trial. Moreover, frequent losses caused subjects to avoid these decks.

### General Discussion

This study has demonstrated that the deck (iB) with frequent losses (punishments) can help subjects avoid having to make repetitive choices regarding the same deck. Accordingly, under uncertainty, decision makers cannot suffer too many losses (i.e. punishments) in a certain deck while attempting to understand the final benefits (rewards).

If the study of Bechara *et al.* (2000) [14] was predictive, the normal decision makers would be sensitive to future consequences. Normal decision makers should subsequently choose the advantageous decks(iA and iB)in both versions of *iIGT* in this study. However, the normal subjects obviously chose the advantageous decks iA, yet avoided the advantageous iB in both stages. Notably, our results demonstrated that frequent small punishments override infrequently large rewards in the *iIGT*.

However, many behavioral-analysis studies have demonstrated that choice patterns in the concurrent reinforcement experiment mainly follow the frequency, magnitude and ratio of reward for each choice [64], [65], [66], [67], [68], [69], [70]. Therefore, these behavioral studies largely support the findings of this study regarding selection under uncertainty.

If the experimental findings from this study are applied, normal decision makers lack the ability to integrate their somatic markers across (uncertain inter-stimulus-interval) trials for specific decks and then hunch the final outcome. Restated, if the SMH holds, an “efficient and precise” internal bank is established to assist in decision makers' survival in social environments. However, life seldom meets expectations, especially when facing uncertainty [71], [72]. Conversely, an immediate response to quick and uncertain events may be easy for humans to survive to the next moment. According to many biologists, many social animals (e.g., bat or chimps) use a popular strategy (i.e. “tit for tat”) to cope with complex and uncertain social situations. Correspondingly, this strategy benefits the “species” in the long run [71], [72], [73], yet does not do so for the “individual”, unlike the SMH. Some studies on altruistic punishment in neuroeconomics also confer with this perspective [74], [75], [76], [77]. Therefore, causing an organism to suffer frequent small losses (punishments) in order to achieve an unexpected large long-term gain (reward) under uncertainty is extremely difficult.

### Conclusions

Bechara *et al.* [1] developed the IGT to verify the critical role of VMPFC in decision making under uncertainty. Moreover, based on the *iIGT*, Bechara *et al.* identified the basic assumption of SMH. However, growing evidence of the PDB phenomenon [15], [16], [17], [18], [19], [20], [21], [23], [24], [25], [26], [30] has challenged the cornerstone of the IGT. The “gain-loss frequency” easily explains the PDB phenomenon in the IGT. Nevertheless, the inverted PDB phenomenon in the *iIGT* has seldom been verified in the literature. This study thus illustrates the mirrored PDB phenomenon by undertaking two double-stage experiments using the *iIGT* and then applying its simplified version. Empirical results indicate that subjects avoided advantageous deck iB in the *iIGT* and the simple version of the *iIGT*. The mirrored PDB phenomenon demonstrates that high-frequency loss prevents decision makers from choosing the good final-outcome deck iB. Consequently, decision makers are more significantly influenced by gain-loss frequency than by final outcome under uncertainty. “No pain no gain” may be an optimized rule for certain circumstances in the long-term dynamic game. Conversely, frequent loss (pain) is intolerable for most individuals and makes long-term gain invisible and inaccessible under uncertainty. The gain-loss frequency may be a critical index of clinical diagnosis for affective disorders [6], [32].

## Supporting Information

Figure S1 **The correlation between monetary value and selection probability of consecutive trials in each deck.** Furthermore, this study conducted a choice-probability analysis after subjects received monetary feedback in each deck. Consequently, the examination revealed that subjects displayed neither increased probability of remaining at the same deck after receiving big gains (e.g. $ +1150, $+250, $+200…) nor decreased probability of choosing the same deck after experiencing losses (e.g. $ −200, $−100, $−50…). The regression analysis indicated that monetary value is not a determinant of choice probability (Deck iA: R^2^ = .0004; Deck iB: R^2^ = .0273; Deck iC: R^2^ = .0042; Deck iD: R^2^ = .0010). Correlation analysis revealed no significant correlation between the gain-loss value and the selection probability of consecutive trials in each deck. Namely, subject choice behavior was lightly influenced by the intensity of gain-loss value. For example, the big gain ($ 250) of deck iA did not increase the consecutive choice probability than that of small gain ($50, $100, $150, $200) and the loss ($ −100).
(TIF)

Figure S2 **The correlation between monetary value and choosing probability of consecutive trials in the iA-iA-iC-iC and iB-iB-iD-iD versions.** Even in the relative simple context, subject choice behavior did not depend on feedback regarding monetary value. Namely, big gains ($ 250, $ 200) rather than small gains ($ 50) or losses ($ −100) of deck iA did not increase the probability of subjects choosing the same deck. Additionally, the other three decks had similar R-square values close to zero (Deck iA: R^2^ = .0011; iC: R^2^ = .0042) (Deck iB: R^2^ = .0181; Deck iD: R^2^ = .0000). The gain-loss value and selection probability of consecutive trials in each deck are not significantly correlated. The slopes of the regression lines of each deck are all close to zero, demonstrating monetary value was less of an influence on the subsequent trial selection. Specifically, subjects choose to remain at the same decks or shift to other decks for reasons unrelated to the monetary value feedback from the last trial. Compared to the low-monetary gain, high-monetary gain did not increase the probability of subjects staying at the same deck. Similarly, compared with the low-monetary loss, high-monetary loss did not increase the probability of subjects avoiding the same deck.
(TIF)
