# Supplementary figures and images for: Mirrored Prominent Deck B Phenomenon: Frequent Small Losses Override Infrequent Large Gains in the Inverted Iowa Gambling Task
Source: PLoS One. 2012 Oct 16;7(10):e47202. doi: 10.1371/journal.pone.0047202 (PMC3473047; doi:10.1371/journal.pone.0047202)

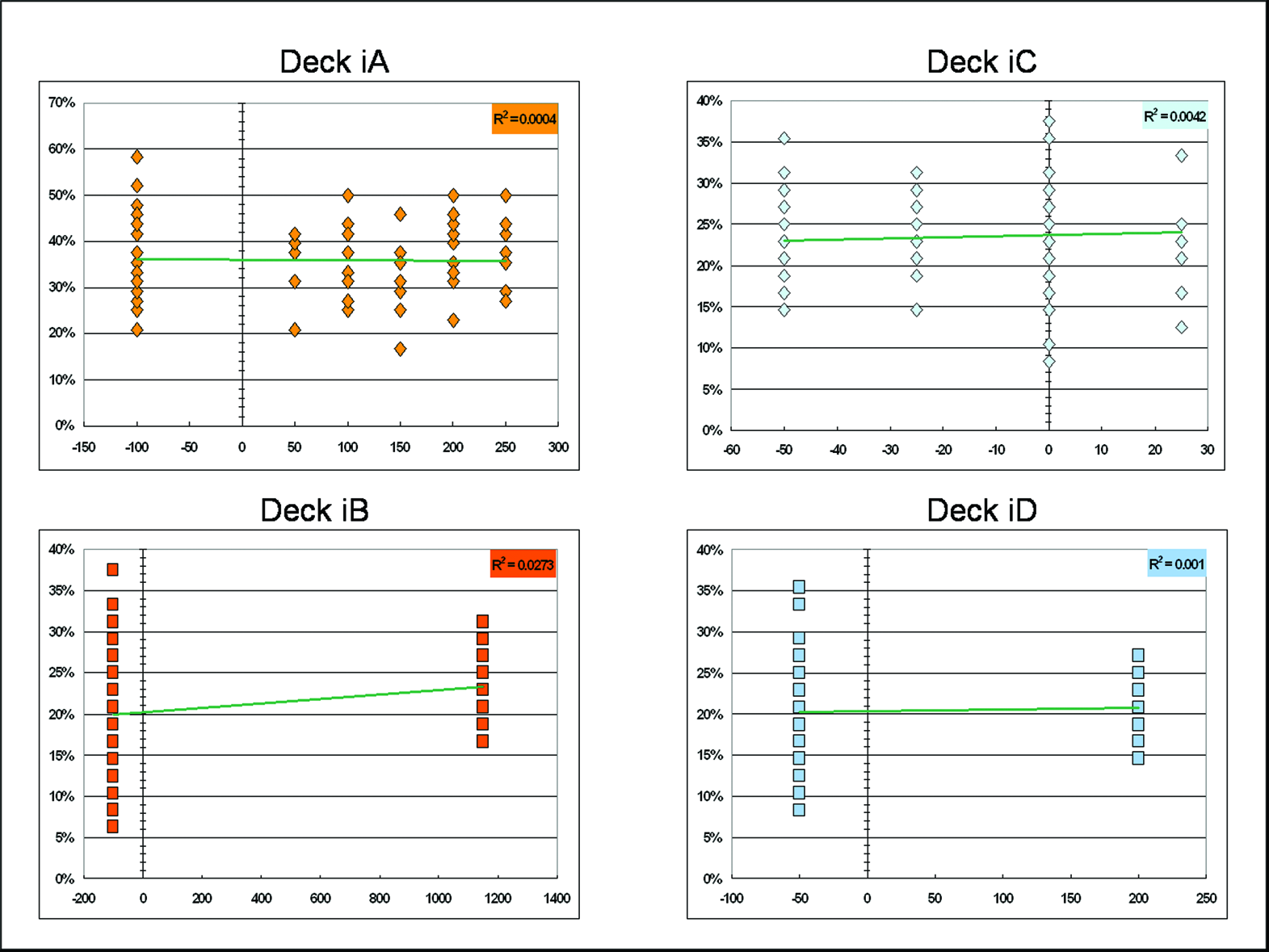

Supplement: Figure S1 — The correlation between monetary value and selection probability of consecutive trials in each deck. Furthermore, this study conducted a choice-probability analysis after subjects received monetary feedback in each deck. Consequently, the examination revealed that subjects displayed neither increased probability of remaining at the same deck after receiving big gains (e.g. $ +1150, $+250, $+200…) nor decreased probability of choosing the same deck after experiencing losses (e.g. $ −200, $−100, $−50…). The regression analysis indicated that monetary value is not a determinant of choice probability (Deck iA: R2 = .0004; Deck iB: R2 = .0273; Deck iC: R2 = .0042; Deck iD: R2 = .0010). Correlation analysis revealed no significant correlation between the gain-loss value and the selection probability of consecutive trials in each deck. Namely, subject choice behavior was lightly influenced by the intensity of gain-loss value. For example, the big gain ($ 250) of deck iA did not increase the consecutive choice probability than that of small gain ($50, $100, $150, $200) and the loss ($ −100). (TIF) [file pone.0047202.s001.tif]

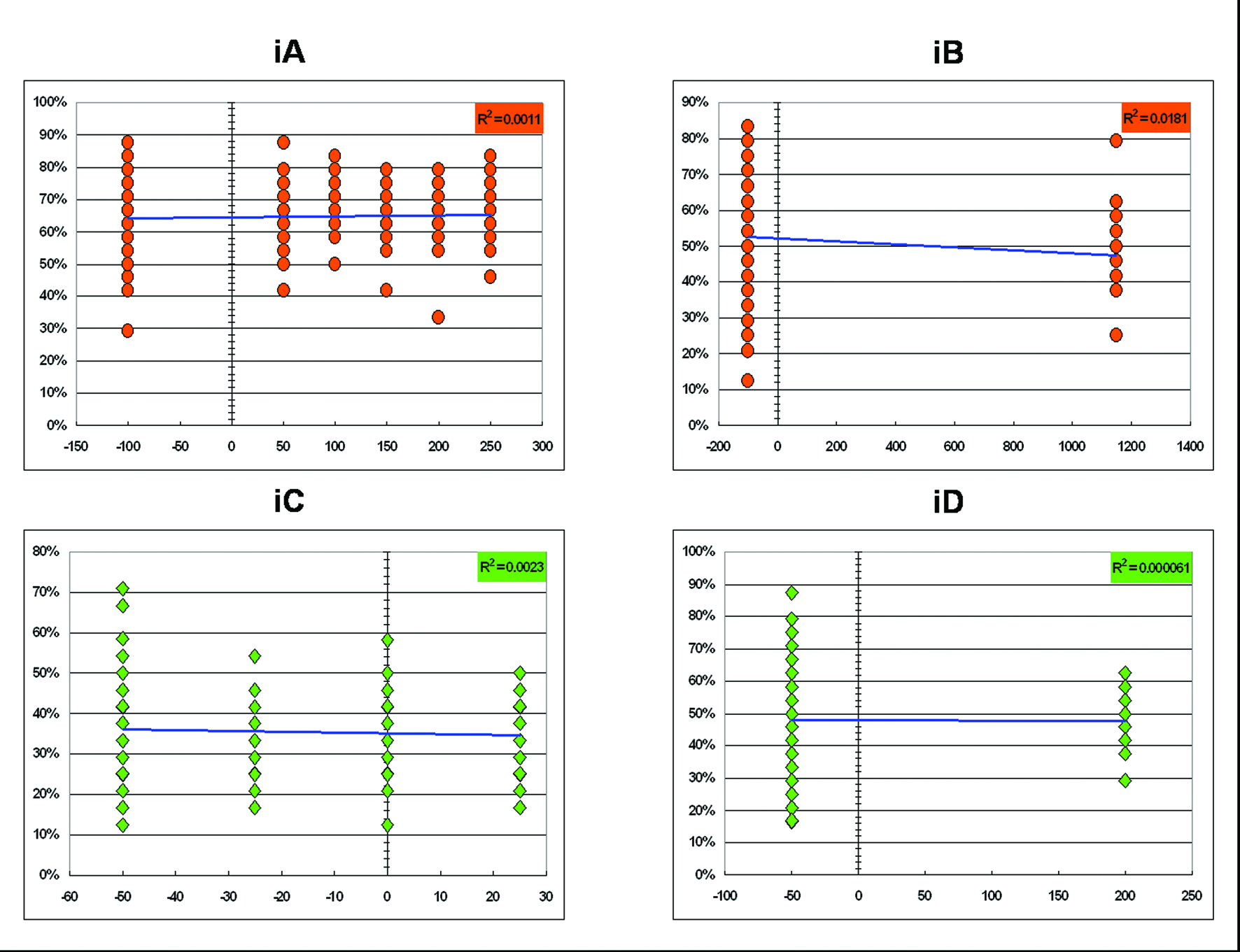

Supplement: Figure S2 — The correlation between monetary value and choosing probability of consecutive trials in the iA-iA-iC-iC and iB-iB-iD-iD versions. Even in the relative simple context, subject choice behavior did not depend on feedback regarding monetary value. Namely, big gains ($ 250, $ 200) rather than small gains ($ 50) or losses ($ −100) of deck iA did not increase the probability of subjects choosing the same deck. Additionally, the other three decks had similar R-square values close to zero (Deck iA: R2 = .0011; iC: R2 = .0042) (Deck iB: R2 = .0181; Deck iD: R2 = .0000). The gain-loss value and selection probability of consecutive trials in each deck are not significantly correlated. The slopes of the regression lines of each deck are all close to zero, demonstrating monetary value was less of an influence on the subsequent trial selection. Specifically, subjects choose to remain at the same decks or shift to other decks for reasons unrelated to the monetary value feedback from the last trial. Compared to the low-monetary gain, high-monetary gain did not increase the probability of subjects staying at the same deck. Similarly, compared with the low-monetary loss, high-monetary loss did not increase the probability of subjects avoiding the same deck. (TIF) [file pone.0047202.s002.tif]
